# A bibliometric analysis and visualization of medical data mining research

**DOI:** 10.1097/MD.0000000000020338

**Published:** 2020-05-29

**Authors:** Yuanzhang Hu, Zeyun Yu, Xiaoen Cheng, Yue Luo, Chuanbiao Wen

**Affiliations:** a School of Medical Information Engineering, Chengdu University of Traditional Chinese Medicine, Chengdu, Sichuan; b College of Acupuncture and TuiNa, Chengdu University of Traditional Chinese Medicine, Chengdu, China.

**Keywords:** bibliometric analysis, CiteSpace, medical data mining, visualization, VOSviewer

## Abstract

**Background:**

Data mining technology used in the field of medicine has been widely studied by scholars all over the world. But there is little research on medical data mining (MDM) from the perspectives of bibliometrics and visualization, and the research topics and development trends in this field are still unclear.

**Methods:**

This paper has applied bibliometric visualization software tools, VOSviewer 1.6.10 and CiteSpace V, to study the citation characteristics, international cooperation, author cooperation, and geographical distribution of the MDM.

**Results:**

A total of 1575 documents are obtained, and the most frequent document type is article (1376). SHAN NH is the most productive author, with the highest number of publications of 12, and the Gillies's article (750 times citation) is the most cited paper. The most productive country and institution in MDM is the USA (559) and US FDA (35), respectively. The Journal of Biomedical Informatics, Expert Systems with Applications and Journal of Medical Systems are the most productive journals, which reflected the nature of the research, and keywords “classification (790)” and “system (576)” have the strongest strength. The hot topics in MDM are drug discovery, medical imaging, vaccine safety, and so on. The 3 frontier topics are reporting system, precision medicine, and inflammation, and would be the foci of future research.

**Conclusion:**

The present study provides a panoramic view of data mining methods applied in medicine by visualization and bibliometrics. Analysis of authors, journals, institutions, and countries could provide reference for researchers who are fresh to the field in different ways. Researchers may also consider the emerging trends when deciding the direction of their study.

## 1 Introduction

Data mining, also known as database knowledge discovery, is a powerful method to extract knowledge from data, which is supposed to be able to handle various data types in all formats.^[1]^ Medical data mining (MDM) is defined as an extraction of implicit, potentially useful and novel information from medical data to improve accuracy, decrease time and cost, and construct decision support system with the aim of health promotion. Driven by the rapid development of science and technology, the hospital information construction is becoming more and more perfect, and the medical data storage volume is getting larger. The research on MDM is growing fast, and the application of data mining in medicine is most used by data mining developers and academic researchers.^[2]^ The study of MDM is started from last decades and now it is in a teenage period. How to effectively use the data analysis method to mine the high-value information contained in the massive medical data, and then realize the knowledge discovery, and how to serve the clinical practice and scientific decision-making in hospitals are the great concerns in the field of MDM.

Bibliometrics is the cross-disciplinary science of quantitative analysis of all knowledge carried by mathematical and statistical methods.^[3]^ In light of bibliometric methods, the latest advances, leading topics, current gaps in a certain field of research discipline can be drawn vividly as well as geographically, and it is becoming an import research method for assessing national and international research productivity, international cooperation, citation analysis, research trends, and development of specific fields. At present, many bibliometric analysis methods and tools like CiteSpace and VOSviewer have been developed to help researchers in different field construct knowledge maps, evaluate the collective state of thought about a subject, and identify hotspots in a research field.

In this paper, we use free available software VOSviewer 1.6.10 to carry out the visualized map, and CiteSpace V to generate diagrams and calculate the betweenness centrality score. The literature on the application of data mining technology in the medical field from the Web of Science database has analyzed to provide a macroscopic overview on the main characteristics of MDM publication. And clear informative pictures presented in this paper demonstrate the research achievements in the domain of the MDM, which could help researchers and practitioners identify the underlying impacts from authors, journals, countries, institution, references, and research topics. Although this work is not structured as an exhaustive analysis of MDM-related literature, it does illustrate the utility of bibliometric techniques for exploring hidden knowledge spaces.^[4]^

## 2 Data and methods

### 2.1 Data collection

The literature data involved in this study are retrieved form the core collection of Web of Science (WOS).^[5]^ The WOS is one of the most comprehensive bibliographic sources available, and provides users an online access port to a number of resources, including massive citation databases, but not all journals or articles are indexed.^[6]^

For the purpose of this paper, we are interested in exploring the knowledge domain associated with “medical data mining,” and use “medical data mining” as the search term in the WOS database, the literature type is defined as “all types.” For assuring the quality of data, a manual review on search results is adopted in Endnote X9 to remove the unrelated papers, and the CiteSpace function, Alias^[7]^ is used to identify and correct all duplicate values in the databases. Finally, 1575 documents are saved as “Plain Text” with “Full Record and Cited References.” And the timespan is from January 1, 2011 to August 28, 2019, which including information on title, author, keywords, abstract, journal, and year. These records are then exported to CiteSpace and VOSviewer for subsequent analysis, and 5 document types are found (Table 1).

**Table 1:**
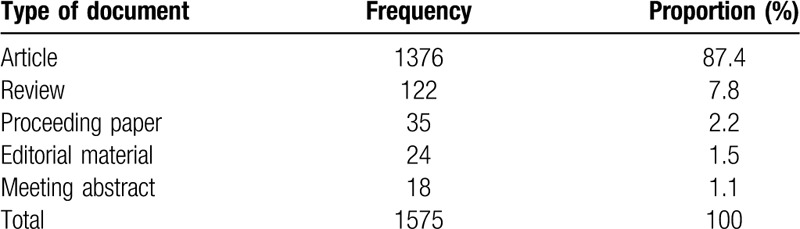
Types of retrieved documents.

### 2.2 Analysis methods

Bibliometric analysis offers additional data statistics including author, affiliation, and keywords. In this context, the items of analysis used in the study are detailed like co-authorship, journal analysis, citing, keyword analysis, geo/location collaboration, co-occurrence, and betweenness centrality score.

Betweenness centrality is a way of detecting the amount of influence a node has over the flow of information in a graph, and often used to find nodes that serve as a bridge from one part of a graph to another. For users, betweenness centrality can make it easier to identify pivotal points, which are highlighted in the display with a purple ring in order to stand out in a visualized map.^[8]^ At the document level, the importance of each document in a co-citing map can be partially evaluated by the indicator betweenness centrality.^[9]^

And we use Price's law (1) to measure core authors. Price's law defines that, 50% of the work is done by the square root of the total number of people who participate in the work.^[10]^ 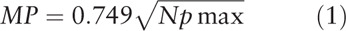


## 3 Findings

### 3.1 Publication growth trend

**Figure 1:**
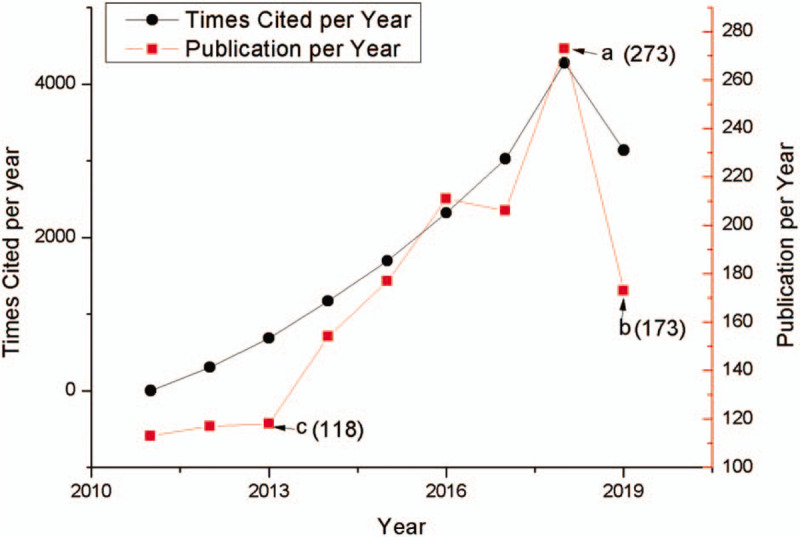
Annual publications and citations of MDM based on WoS. (a) The publication in 2018; (b) the publication between January 1 and August 28, 2019; (c) the publication in 2013.

The quantity of the publication is an important index that reveals the development trends of scientific research. Figure 1 depicts a chronological view on volume of articles published and cited on MDM.

### 3.2 Productivity and connectivity

The core authors in the academic community are the important internal strength to promote the development of the discipline,^[11]^ and researchers can identify potential collaborators and understand how their own research fits in MDM research.

There are 6258 authors in the field of MDM, and Table 2 shows the 20 most productive scholars in the MDM research worldwide. These authors have successfully established broad cooperation with researchers in other countries. Among them, the application of data mining methods in the field of medicine can be divided into a few core research teams, and 1 important team is LEWIS P, GANO M, MORO PL, SHIMABUKURO TT, and STEWART B, which focuses on vaccine adverse event reporting, outlier detection and disease risk-factors.^[12,13]^ As for individual researcher, the most productive author is SHAN NH, who based at the Stanford Center for Biomedical Informatics Research (BMIR) and studies pharmacovigilance and text mining,^[14]^ then followed by LI L, who based at the Icahn School of Medicine at Mount Sinai and studies systems biology and machine learning.^[15]^ HU YH, based at the National Chung Cheng University, Taiwan, and studies large scale medical data preprocessing approach.^[16]^ WANG S, based at the University of Queensland, Australia, and studies medical database management. For example, 1 of his studies proposed a framework to effectively assigns the disease labels, medical chart, and note data of a patient are used to extract distinctive features.^[17]^ REINER BI is based at the Department of Radiology, Veterans Affairs Maryland Healthcare System, studies medical imaging data analysis and quantifying analysis of uncertainty in medical reporting.^[18–20]^ LIU HF is based at the Division of Biomedical Statistics and Informatics, Mayo Clinic, studies mining drug–drug interaction adverse events and reporting.^[21]^

**Table 2:**
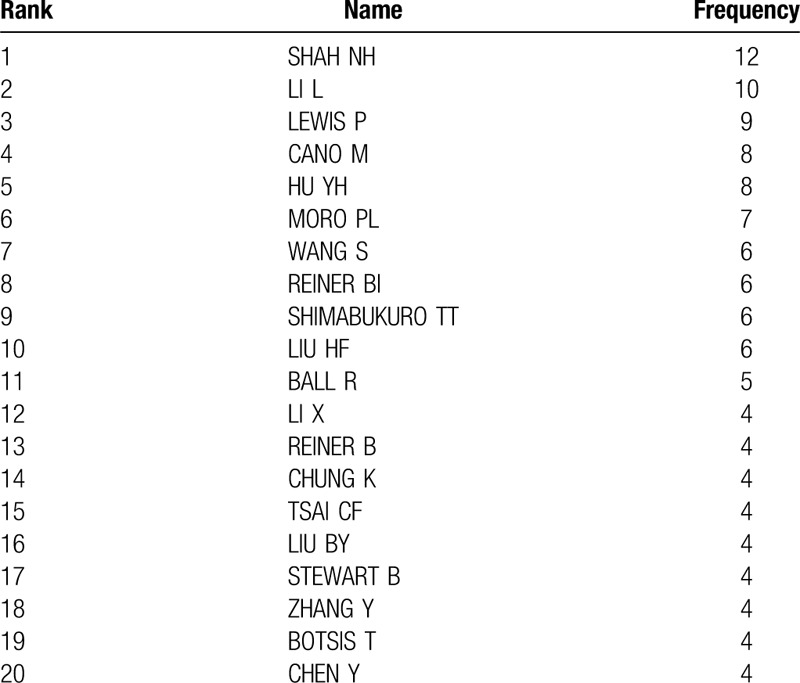
Most productive scholars in MDM research worldwide.

According to Price's law, there are 163 core authors who have published at least 3 papers, accounting for 1.95% of total scholars (less than 50%), which implies that the application of data mining technology in the field of medicine is in the stage of rapid development.

### 3.3 The distribution of institutes on MDM study

**Figure 2:**
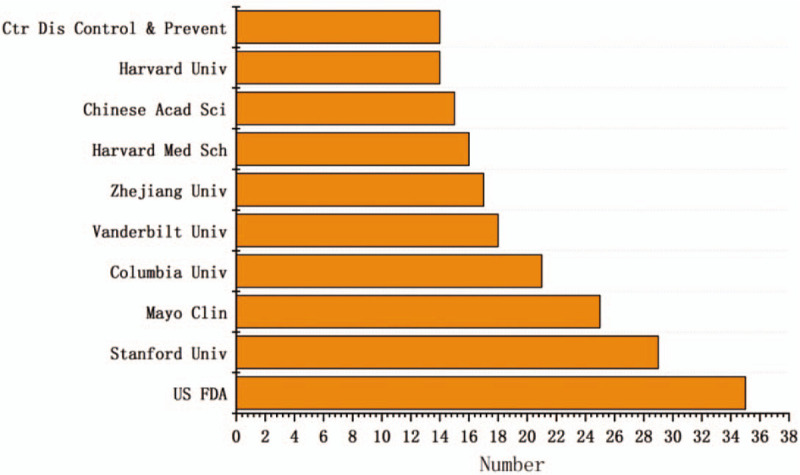
The top 10 institutes with MDM-related publications.

The analysis of MDM research institutions can clarify the core institutions. There are total 2222 organizations and 34 of them produce more than 9 papers. US FDA possesses the greatest number of publications with a total 35 papers, accounting for 3.6% of all publications in this field. At the second position is the Leland Stanford Junior University with 29 publications, then followed by Mayo Clinic, Columbia University in the City of New York, and Vanderbilt University. The top 10 institutes are listed in Figure 2. Among them, 7 institutes are from American and 3 from China. In addition, the 10 institutes totally cover 204 published papers.

### 3.4 Countries/regions cooperation analyses

**Figure 3:**
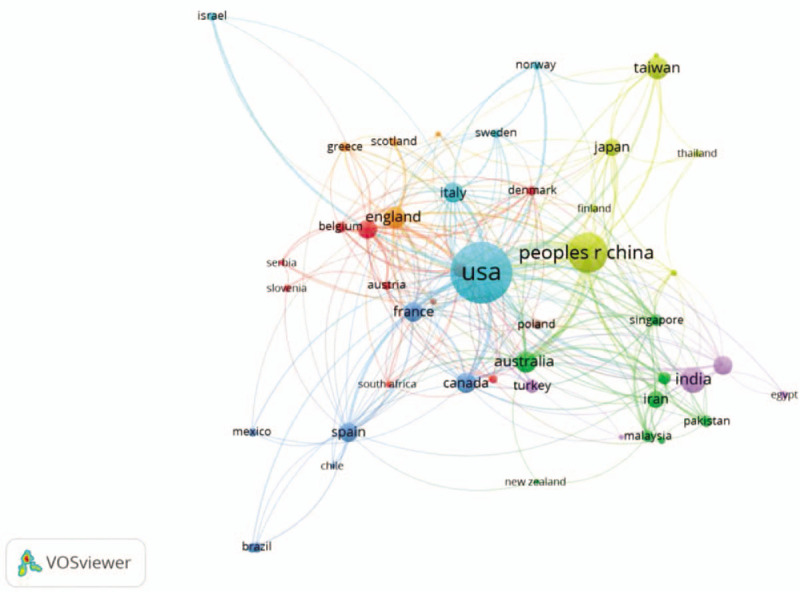
Citation visualization network map of countries/regions based on citation-weights.

Based on the bibliographic data collected from WOS, the countries co-authorship network visualization map is created by VOSviewer (Fig. 3); the minimum document threshold of a country is 5 and there are 47 countries out of 91 listed are visualization items. Specifically, the USA is identified as the country with the largest amount of studies (559, 1/3th of the total publications), followed by China (238), India (102), Taiwan (76), Australia (72), England (65), and Italy (63), because the leading groups of research and practitioners in MDM are located in the above countries or areas.

### 3.5 Journals publishing on MDM

Through the analysis of journals, we can have a better understanding about the structure of core journals in the field of MDM. In total, there are 1575 publications in 650 different journals, but 18. 77% (n = 122) journals have published more than 3 papers. A list of the top 10 most productive journals on MDM research is provided in Table 3, and the top 3 most productive journals are *Journal of Biomedical Informatics*, *Expert Systems with Applications*, and *Journal of Medical Systems*.

**Table 3:**
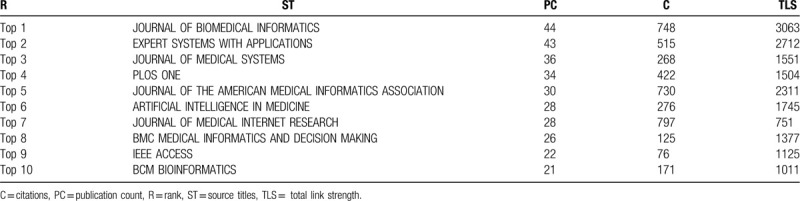
Top 10 most productive sources.

**Figure 4:**
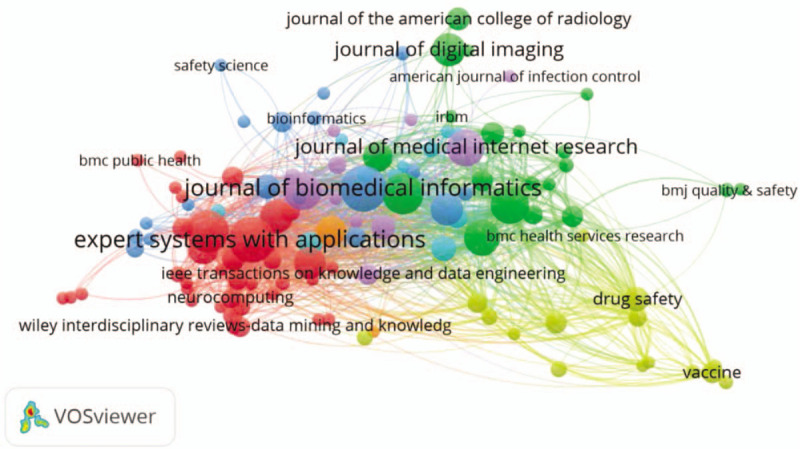
The visualization map of journal publications.

As shown in Figure 4, the size of the nodes represents the publication amount of a journal, and the color of the nodes demonstrates the subdomains of the MDM research. We use VOSviewer to plot the journal co-citation network and generally the smaller the distance between 2 nodes is, the higher of the citation frequency is. In Figure 4, the largest set of the connected items consists of 119 items and some of the 122 items in the network are not connected to each other. It is manifest that all these journals are divided into 5 clusters; the highly cited journals in the blue cluster are representing biomedical information journals, which starts with *JOURNAL of BIOMEDICAL INFORMATICS*. The red cluster represents Management integration journals, which contains *EXPERT SYSTEMS with APPLICATIONS.* The green cluster contains journals on imaging and genetics, starts with *JOURNAL of DIGITAL IMAGING and FRONTIERS IN GENETICS.* The yellow cluster represents drug and vaccine journals. The last purple cluster represents software engineering journals, which contains *IEEE TRANSACTIONS ON VISUALIZATION and COMPUTER GRAPHICS*. For MDM researchers, IEEE is the world's largest technical organization dedicated to advancing computer technology for the benefit of medicine, and it is important to follow IEEE publications and conferences to keep abreast of their latest research status.

### 3.6 Keyword analysis with co-occurrence

**Figure 5:**
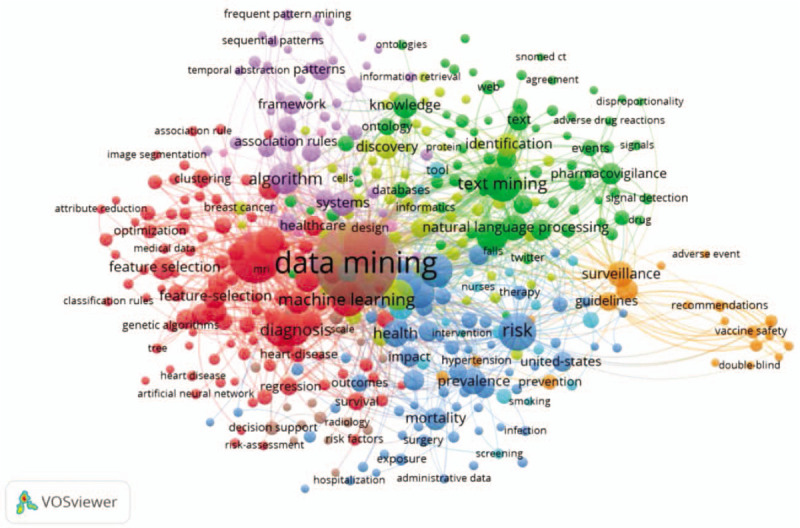
Co-keyword network visualization based on occurrences.

Keywords are nouns or phrases that reflect the core content of a publication.^[22]^ The co-occurrence network of keywords reflects the research hotpots. In this part, the content is studied by analyzing the distribution of keyword. The bibliometric data shows that there are 6992 keywords involved in this research. To illustrate the research hotspots in MDM, keywords co-occurrence threshold is set as 5 and 413 items are brought into visualization (Fig. 5), which is constructed by the VOSviewer. In the network, visualization items are represented by their label and a circle. The size of the label and the circle of an item are determined by the weight of the item. The higher the weight of an item is, the larger the label and the circle of the item are.^[23]^ The color of an item is determined by the cluster to which the item belongs and lines between items represent links in Table 4. In addition to keyword “data mining,” the keywords “classification (790)” and “system (576)” have the strongest strength. The co-keyword network in Figure 5 clearly illustrates 6 distinct clusters and each of them represents a subfield of MDM. As shown in the red cluster (cluster 1, left, 102 items) which contains keywords such as classification, diagnosis, feature selection, artificial neural network, support vector machines, etc, these studies use algorithms to find patterns that make early detection, prediction of the disease, and proper treatment easier.^[24–26]^ The green cluster (cluster 2, upper right, 75items) is associated with text mining, electronic health records, pharmacovigilance, adverse drug reactions, signal-detection, natural language processing, etc, focusing on the main domain of “medical text and language mining.” Studies claim that text mining can be applied to extract useful adverse drug event-related information, form multiple textual sources like electronic health records and improve adverse drug event (ADE) detection and assessment.^[27–29]^ Next, in the blue cluster (cluster 3, bottom right, 67 items), keywords like risk, prevalence, hospitalization, mortality, adverse events, disease, infection, etc, are associated with disease topics.^[30–33]^ In this cluster, the machine learning approaches are mainly applied to disease risk model.^[34]^ In the yellow cluster (cluster 4, 58 items), keywords like discovery, identification, genes, protein, informatics, breast cancer, patient, etc, concentrate on the aspect of “drug discovery.”^[35,36]^ In the orange cluster (cluster 5, lower right, 20items) comprised keywords like surveillance, vaccine safety, vaccine adverse event reporting system (VAERS), recommendations, etc, are more concerned with “medical safety.”^[37,38]^ The last purple cluster (cluster 6, upper left, 44items) gathers keywords like framework, patterns, frequent pattern mining, methodology, decision support system, clinical pathways, etc, mainly concerning “medical mining system.”

**Table 4:**
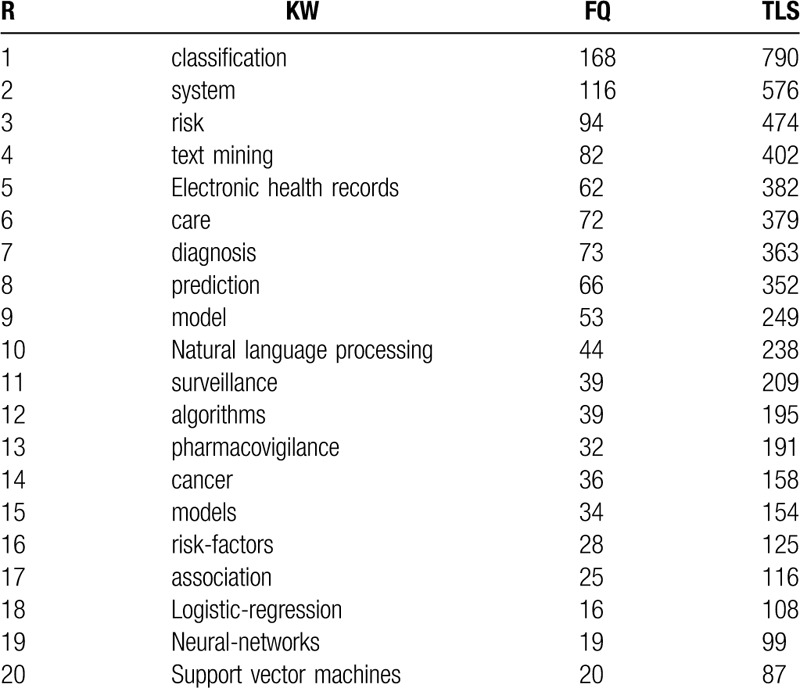
The top 20 keywords of the MDM publications.

We use CiteSpace to generate cluster labels, usually the LLR (log-likelihood tests) algorithm gives the best result in terms of the uniqueness and coverage. There we have got 11 clusters based on LLR: #0 extraction system, #1 deep learning, #2 knowledge-based systems, #3 decision tree, #4 drug discovery, #5 medical imaging, #6 gene ontology, #7 instance selection, #8 hepatitis, #9 vaccine safety, and #10 clinical decision support.

Table 5 shows which keywords have the strongest bursts and which period of time the strongest bursts takes place (settings: years per slice: 1, node types: keyword, top N per slice: 50). The red part represents the period when the citation burst has happened. “Bioinformatics” is the first keyword proposed in MDM research. “Association rule” has the longest period of burst from 2011 till 2015. The keyword “clinical decision support,” “feature selection,” “children,” “impact,” and “quality” are the nearest hot-spot keyword in the burst. Keyword burst also shows that the theme of the study changes quickly as time goes on, and many branches of MDM research are synchronously thriving (Table 6), like “reporting system,” “precision medicine,” and “inflammation.”

**Table 5:**
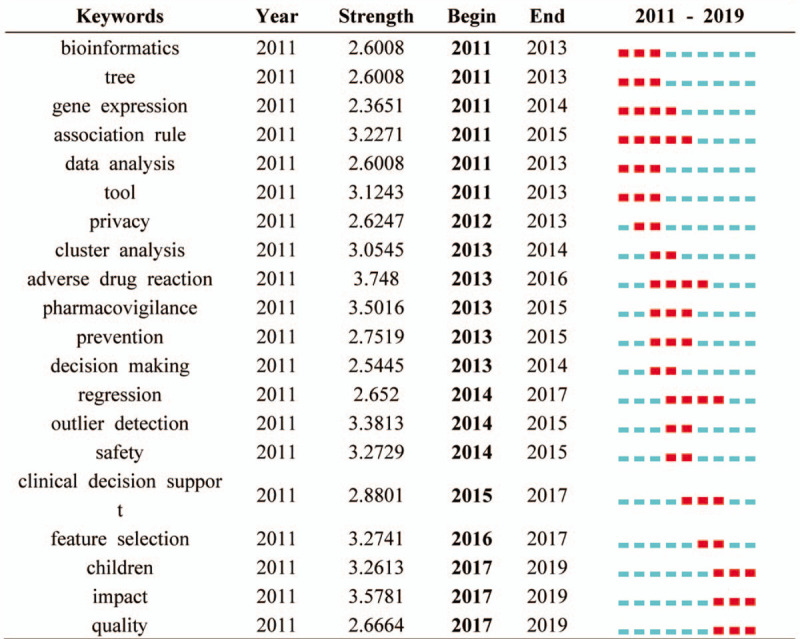
Top 20 keywords with the strongest citation bursts.

**Table 6:**
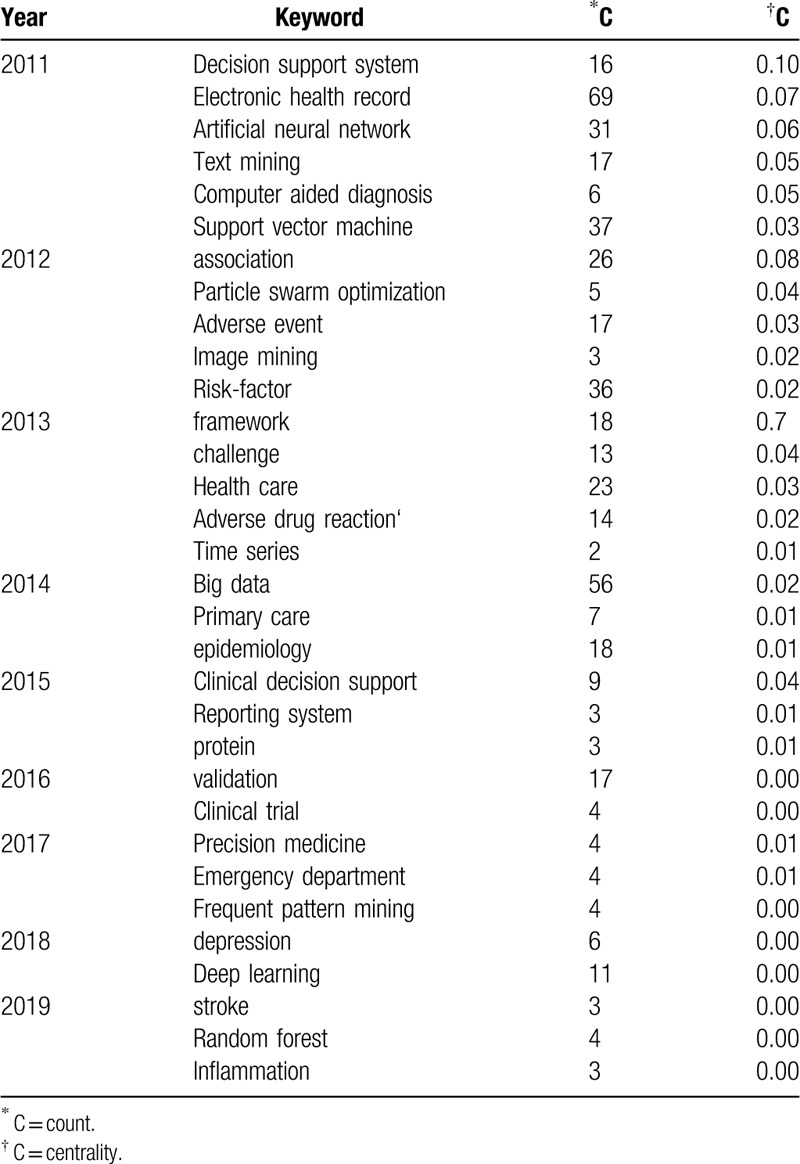
Analysis of the keywords and centrality of MDM.

### 3.7 Most cited papers

Citation analysis is one of the parameters for assessing the quality of research. Table 7 lists the total citations, titles, authors, and publication years of the top 20 most cited papers of MDM. Among these 20 papers, 12 papers receive more than 120 citations and 4 papers receive more than 180 citations. Lambin describes the process of radiomics, and provides a guidance for investigating the standardized evaluation of both the scientific integrity and the clinical relevance of the numerous publishes.^[39]^ Gillies put forward the opinion that converting radiomics image to higher-dimensional data and mining of these data could improve clinical decision support.^[40]^ Parmar proposes a semiautomatic region that can grow volumetric segmentation algorithm, which investigates in terms of its robustness for quantitative imaging feature extraction and uses 14 feature selection methods and 12 classification methods to examine in terms of their performance and stability for predicting overall survival. The variability analysis indicates that the choice of classification method is the most dominant source of performance variation.^[41,42]^ Besides that, Jensen proposes that integrating electronic health records (EHR) data with genetic data will give a finer understanding of genotype–phenotype relationship.^[29]^ In sum, these articles mentioned above showed the part application about data mining methods in MDM from different aspects.

**Table 7:**
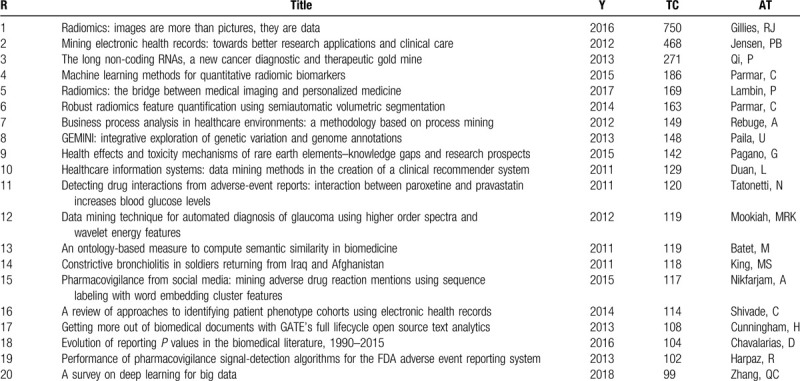
The 20 most cited documents in MDM according to WOS.

### 3.8 Co-cited papers in the field of MDM

**Figure 6:**
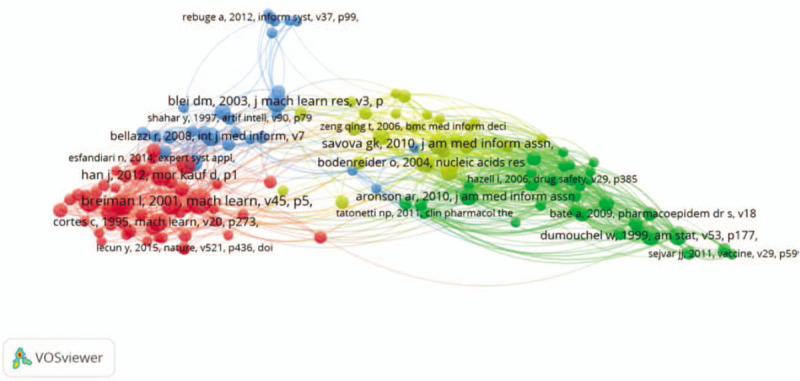
Cited references network of MDM.

The co-citation analysis assesses if articles are cited together and their corresponding frequencies and scales. If 2 articles are both cited as references in another article, then those 2 papers have a co-citation relationship. In this essay, VOSviewer is used to build a co-citation paper network for MDM research, the network of articles represents the intellectual basis of the field (Fig. 6). The MDM papers identified here cite collectively 55,175 unique publications, among them, 139 papers which have been co-cited more than 10 times are analyzed. As the visualization illustrated, each cluster has a color that indicates the group to which the cluster is assigned. We can see that all these papers are divided into 4 clusters. The red cluster, in terms of citations received, is led by L Breiman's article (*Breiman, 2001, Machine Learning: Random Forests*),^[43]^ followed by C Cortes (*1995, Support-Vector Networks*),^[44]^ Esfandiari (*2014, Knowledge discovery in medicine: Current issue and future trend*),^[1]^ LeCun Y (*2015, Deep learning*),^[45]^ and Alex Krizhevsky (*2017, ImageNet Classification with Deep Convolutional Neural Networks*).^[46]^ These studies propose some deep learning algorithms like deep convolutional neural network, random forests, and support-vector network. The blue cluster has Rojas E (*2016, Process mining in healthcare: A literature review*)^[47]^ and Rakesh Agrawal (*1993, Mining association rules between sets of items in large databases; 1994, Fast Algorithms for Mining Association Rules and 1995, Mining sequential patterns*),^[48–50]^ which studies association rules and processes mining for healthcare processes. The yellow and green are tightly connected to each other, indicating shared relevant literatures compared to the rest of the network. The yellow cluster contains Savova GK (*2010, Mayo clinical Text Analysis and Knowledge Extraction System*),^[51]^ Nat Genet (*2000, Gene ontology: tool for the unification of biology. The Gene Ontology Consortium*),^[52]^ and Olivier Bodenreider (*The Unified Medical Language System (UMLS): integrating biomedical terminology*)^[53]^ studying the medical mining system. George Hripcsak (*2013, Next-generation phenotyping of electronic health records*) and Jensen Peter B (*2012, Mining electronic health records: towards better research applications and clinical care*) apply text mining in electronic health records for providing assistance to the physician.^[29,54]^ The green cluster has DuMouchel W (*1999, Bayesian data mining in large frequency tables, with an application to the FDA spontaneous reporting system: Discussion*),^[55]^ Aronson AR (*2010. An overview of MetaMap: historical perspective and recent advances*),^[56]^ P LePendu (*2013, Pharmacovigilance Using Clinical Notes*),^[28]^ Bate A (*2009, Quantitative signal detection using spontaneous ADR reporting*),^[57]^ and David C. Classen (*2011, ’Global Trigger Tool’ Shows That Adverse Events In Hospitals May Be Ten Times Greater Than Previously Measured*) studying bayes model, adverse medical events, and biomedical information.^[58]^

A citation burst has 2 attributes: the intensity of the burst and how long the burst status lasts. Table 8 shows references with the strongest citation bursts across the entire dataset during the period of 2011 to 2019. The first burst article is Marylyn D. Ritchie (2012–2013, 2. 6377), who studies EMR-linked DNA bio-repository to detect known genotype–phenotype associations; the result demonstrates that phenotypes representing clinical diagnoses can be extracted from EMR systems.^[59]^ The strongest strength is Mohammed Saeed (2016–2017, 7. 2014), who develops an intensive care unit research database and applies automated techniques to aggregate high-resolution diagnostic and therapeutic data from a large, diverse population of adult intensive care unit patients.^[60]^ Followed by SEJVAR JJ and MARTIN D (both 2015–2016, 6. 4047), SEJVAR JJ provides the case definitions and guidelines for the standardized collection and assessment of information about Guillain Barré syndrome (GBS) and Fisher syndrome (FS).^[61]^ MARTIN D focuses on using data mining methods to find a novel safety signal for vaccine safety monitoring.^[62]^ The nearest burst reference is HRIPCSAK G (2016–2019, 3. 2683) and Wei CH (2017–2019, 3. 8182). HRIPCSAK G focuses on next-generation phenotyping of EHR for the complex, inaccurate, and frequently missing of the EHR data; and Wei CH describes PubTator, an automated text mining tool for curating knowledge from biomedical literature into structured databases.^[54,63]^

**Table 8:**
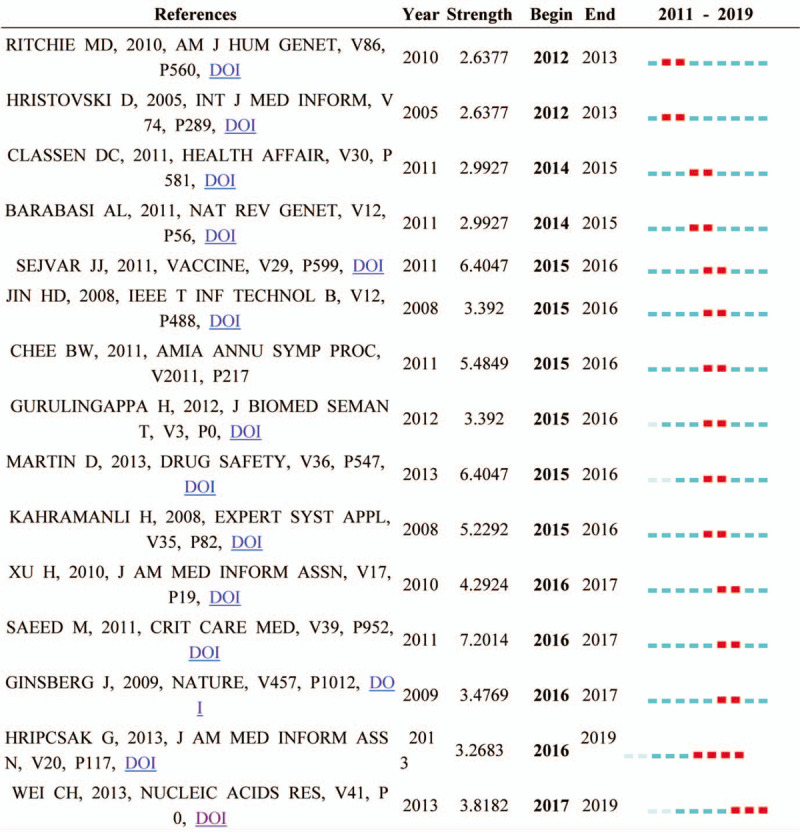
A total 15 references with the strongest citation bursts over the period between 2011 and 2019 are shown.

## 4 Discussions and conclusions

### 4.1 Findings and discussions

The knowledge map of MDM was visualized by information visualization software Citespace and VOSviewer based on the literature retrieved from WOS for 2011 to 2019 years. Through the author analysis, journal analysis, country analysis, institution analysis, co-cited references network analysis, co-occurrence keywords network analysis and burst keywords analysis, the research achievements, and potential impacts of MDM have been identified in a multipurpose and comprehensive way. Some interesting results concerning the MDM-related publications can be summarized as follows:

First, from the research analyze of publications, the annual number of published and cited papers have gradually increased during the last decades based on the data from WOS. Most notably the publication output on MDM cities has increased exponentially since 2013, which represents the kick-off of MDM due to the success of application of data mining technology in other fields. There is a growing interest in the researches related to the MDM, which corresponds to the urgent need for discovering medicine knowledge, assisting physicians, improving public health, and supporting patients.^[64–66]^

Second, in terms of institutes, the US FDA has the highest number of publications. The USA has 7 institutes among the top 10 institutes with regard to the number of MDM-related publications, which implies that the USA is the bellwether in this field and *JOURNAL OF BIOMEDICAL INFORMATICS* ranks first among the journals.

Third, keywords burst is an indicator of a most active area of research, which refers to these keywords increase particular attention from the related scientific communities in a certain period of time.^[67]^ Based on the co-keyword network and burst analysis, we have found that there are some new study trends like extracting information from the text of electronic medical records (EMR) and mining genetic data. Also, the patient-centered model is an inevitable trend in future medical development, and there are great discussions about precision medicine.^[68–70]^ The big-data revolution will vastly improve the granularity and timeliness of available epidemiological information with hybrid systems augmenting.^[71]^ And data mining analysis is the key to precision medicine treatment.^[72]^ Denny also mentions that natural language processing methods to process narrative text data may be needed.^[73]^ Wagner provides a tool (DGIdb, www.dgidb.org) and Pinero developed a platform DisGeNET (www.disgenet.org) for mining the druggable genome for precision medicine hypothesis generation.^[74,75]^ In addition to the genome data, medical images are also the important data sources of MDM. Radiomics has been defined as the conversion of images to minable data, which benefit to yield quantitative image-based phenotypes for data mining with other-omics for discovery (i.e., imaging genomics) or yield predictive image-based phenotypes of disease for precision medicine.^[76]^ In the future, since the continuous development of computer software and hardware, the application of data mining technology in radiology may allow radiologists to further integrate their knowledge with their clinical colleagues in other medical specialties, and promote the development of precision medicine. Also, many medical mining systems could help physicians in daily clinical practice, like improving diagnosis accuracy,^[77]^ reducing diagnosis time,^[78]^ precisely providing quantified temporal order information of critical medical behaviors in clinical pathways, and reducing errors in medicine.^[79,80]^

Fourth, the research areas can be divided into clinical application (including screening, diagnosis, treatment, prognosis, monitoring, and management) and data mining approaches (classification, regression, clustering, prediction, association rule mining, and hybrid).^[81–85]^ The clinical support system can be used to in several conditions such as emergency situation, shortage of physicians, and to decrease human errors. The algorithms that are applied in medicine such as logistic-regression, decision tree, neural-networks, support vector machines (SVM), and association rule. Time series and random forest algorithms are the most popular. But each data mining algorithm has its advantages and disadvantages. There are some common strength of the data mining techniques like suitable computational accuracy and ability to handle complex relationship among different features.^[86]^ Besides, each of them has its own strength like the simplicity and comprehensibility of decision tree, popularity, and ability of neural-networks in general model extraction, association rule is suitable for describing frequent patterns among dataset; k-means is easy to implement and understand, and SVM is efficient.^[87–89]^ Despite the advantage, the limitation also should be considered like time consuming and inability to support for large dataset. And each data mining algorithm has its own limitation, as an example, in SVM the generated models are a black box and it is designed essentially for binary classification; in random forest, it cannot estimate values of the variable outside the range of the training data; and in K-Means, it do not explain why and how these samples are grouped into a cluster. Data mining algorithms are capable to obtain valuable knowledge form raw dataset, but models are too complex to understand and interpret by human experts especially in black-box phenomenon. The final goal of modeling in medicine is providing understandable knowledge for physicians to conduct care strategies, so for overcoming this interpretability problem, extraction of rules and visualization could be applied.

Fifth, the number of times an article cited as a reference in another article reflects its scientific impact. And the citation can determine the distribution of the most influential literature in the field of MDM. Among the top 20 citation publications, 5 articles are published in 2011, 10 articles are published from 2012 to 2015, and 5 articles are published after 2016. Because high degree of cooperation with other countries and regions, USA is the most active (documents: 11) and influential (citations: 2566) country and ranks first, which indicates that the United States is the central region of MDM research, then followed by Canada (documents: 4, citations: 918) and Netherlands (documents: 3, citations: 742). China ranks fourth, which had 2 publications and had a citation of 456, shows that the degree of international academic cooperation is not as close as that of the USA. China should pay attention to the scientific of papers published and strengthen cooperation with other countries and regions in the future MDM study.

### 4.2 Limitations and future outlook

Although we have review the papers of MDM and demonstrate the research achievements and the potential impacts according to the authors, journals, countries, institution, references, and research topics, the limitation of this study should be addressed:

(1) The substantial works have published in MDM field, but it is impossible to discuss all of them in a single work. This type of research depends on bibliometric datasets and the datum collection is limited the WOS. In order to have a better understanding for MDM relevant research, better and bigger datasets are needed.
(2) In this study, we only focus on publications between 2011 and 2019 that mentioned MDM in the database which are publicly available on the website, the authors admit the possibility of missing some import research publications on MDM, and some relevant records could be missing, if the query phrases used for topic searches did not match some records. We are hoping that future studies covering longer time period will shed more light on the field, researchers, and publications. We hope this paper will make it possible to explore previous works by visualization and bibliometrics to provide guideline for researchers who are new to the field.

However, there are also other challenges such as structural EMR data, signal, radiomics image, integration with the hospital workflow, and lack of data mining package for medical domain. Noise and missing value also are common challenges in MDM. For process mining in healthcare, there are no portable solutions for all different hospital environments, and lack of a visualization tool of the process models as well as a great reliance on experts.^[90]^ Medical data mining framework could be described as 5 steps:

(1) medical problem understanding;
(2) medical data preview;
(3) discovering relations between data;
(4) extracting relations to be a model;
(5) verification of extracted model by background knowledge.

For the mining framework, although there are some standards such as ICD-10 for disease information integration (WHO),^[87]^ appropriate collection and transmission standards are still absent.

## Author contributions

**Conceptualization:** Yue Luo, Chuanbiao Wen.

**Data curation:** Yuanzhang Hu.

**Formal analysis:** Yuanzhang Hu, Zeyun Yu.

**Methodology:** Yuanzhang Hu, Xiaoen Cheng.

**Project administration:** Chuanbiao Wen.

**Supervision:** Yue Luo, Xiaoen Cheng.

**Writing – original draft:** Yuanzhang Hu, Zeyun Yu.

**Writing – review & editing:** Xiaoen Cheng, Yue Luo, Chuanbiao Wen.

## Correction

Dr. Xiaoen Cheng's name was misspelled in the original publication as Xiaoen Chen. This has since been corrected.
